# A computerized tablet system for evaluating treatment of essential tremor by magnetic resonance guided focused ultrasound

**DOI:** 10.1186/s12883-017-0856-8

**Published:** 2017-04-17

**Authors:** Fred Tam, Yuexi Huang, Michael L. Schwartz, Tom A. Schweizer, Kullervo Hynynen, Simon J. Graham

**Affiliations:** 1 Physical Sciences Platform, Sunnybrook Research Institute, Mailroom S604, 2075 Bayview Avenue, Toronto, ON M4N 3M5 Canada; 2 Department of Surgery (Neurosurgery), Sunnybrook Health Sciences Centre, Toronto, Canada; 3 Division of Neurosurgery, Department of Surgery, University of Toronto, Toronto, Canada; 4 Keenan Research Centre for Biomedical Science of St. Michael’s Hospital, Toronto, Canada; 5 Department of Medical Biophysics, University of Toronto, Toronto, Canada

**Keywords:** Essential tremor, Movement disorders, Magnetic resonance imaging, Magnetic resonance guided focused ultrasound, Tablet, Drawing

## Abstract

**Background:**

Transcranial magnetic resonance guided focused ultrasound is an emerging technology under evaluation for treatment of essential tremor, a prevalent movement disorder. A qualitative evaluation is performed by a clinician periodically during the procedure to maximize treatment effects and minimize adverse effects. The present work demonstrates a magnetic resonance-compatible method to enable more precise, quantitative measurement of tremor severity.

**Methods:**

Tremor severity was measured in 12 patients pre-, post-, and intra-operatively, using a magnetic resonance-compatible tablet and a computerized adaptation of drawing tasks from the widely-used Fahn-Tolosa-Marin Tremor Rating Scale. Tremor metrics based on spectral analysis were calculated for each drawing and compared using Wilcoxon signed rank tests.

**Results:**

Tremor metrics in the dominant (treated) hand were significantly and consistently lower post-operatively compared to pre-operatively, but there was no significant difference in the non-dominant (untreated) hand, as expected. Intra-operative metrics were intermediate between pre- and post-operative metrics.

**Conclusions:**

Use of the tablet for quantitative tremor measurement was demonstrated pre-, post-, and intra-operatively during treatment of essential tremor, complementing standard qualitative assessment. With additional work, the system has potential to add objectivity to clinical trials and to aid treatment decision-making by providing a metric for optimization during the procedure, which may eventually lead to more optimal treatment. Enhancements and further studies are suggested, and extensions to fMRI studies of essential tremor and Parkinson’s disease are also likely.

## Background

Transcranial magnetic resonance guided focused ultrasound (MRgFUS) is an emerging technology that promises revolutionary new treatments of the brain without open neurosurgery [1]. In the past two decades, advances in ultrasound transducers and computation have enabled delivery of focused energy through the intact skull (“sonication”), while advances in magnetic resonance imaging (MRI) techniques have enabled surgical planning with millimetre spatial resolution, as well as accurate, non-invasive temperature mapping for real-time monitoring during therapy. Of the various brain disorders amenable to treatment by transcranial MRgFUS, there has been much early interest in ablation treatments for essential tremor (ET) [1]. ET is a common movement disorder characterized by persistent kinetic or postural tremor of the upper extremities, with a prevalence of 0.9% overall and 4.6% for individuals over 65 years of age [2]. The affliction is progressive and can lead to significant disability and functional impairment, but at least 30% of ET patients do not respond to established medications [3], while a further 30% of ET patients stop taking their medication [2]. In such patients, ablative transcranial MRgFUS can be used to perform a thalamotomy of the ventral intermediate nucleus, the key cerebellar-motor relay where neuronal oscillations have been linked to ET. Initial clinical trials are demonstrating the safety and efficacy of the approach [4, 5].

In addition to preoperative and postoperative imaging sessions and neurological examination, the typical MRgFUS treatment for such patients is conducted over several hours [1]. The target treatment volume for ablation is identified by anatomical MRI, and multiple sonications are then performed to heat the treatment volume under guidance by real-time MRI thermometry. The latter process is performed slowly and cautiously. First, the sonication temperature is raised to sublethal thresholds that have a temporary and reversible effect on neuronal activity, then ablation is performed if appropriate. Throughout treatment, the patient is examined repeatedly by a clinician for both therapeutic (reduced tremor) and adverse events. In particular, sensory or motor disturbances following sublethal sonications enable the clinician to improve targeting of the thermal lesion, to maximize tremor reduction and minimize permanent side effects, such as paresthesia of the fingers.

Although considerable effort has been directed towards optimizing the ultrasound and MRI technology for transcranial MRgFUS procedures, the behavioral testing of patients during treatment has not yet received the same level of attention. Testing typically involves qualitative evaluation of tremor using simple motor and drawing tasks like those from Parts A and B of the Fahn-Tolosa-Marin Clinical Rating Scale for Tremor [6], with emphasis on testing of the treated arm, and questioning the patient for their subjective report of sensorimotor side effects [4, 5, 7]. This testing can be time consuming as it usually requires a clinician to enter the MRI scanner room, moving the patient table out of the scanner, testing, repositioning the patient in the scanner, and clearing the room. Improvements could potentially be made by supplementing qualitative and subjective assessments by others that are more quantitative and objective. Computerized tests administered from the MRI console are particularly attractive. A digital record of treatment effects can be generated automatically by this means. Even more importantly, the quantitative results from computerized tests may provide more accurate assessment of treatment and side effects, and enable more time-efficient testing during sonications as well as between sonications. These improvements to accuracy and efficiency—potentially leading to enhanced patient outcomes and reduced treatment costs—are predicated on the ability to perform quantitative and computerized behavioral testing within the confines of an MRI system during MRgFUS.

Recently, an MRI-compatible computerized behavioral testing system has been developed [8] that holds promise for transcranial MRgFUS applications. The system, which includes a touch-sensitive tablet similar in form to a digitizing graphics tablet, was originally developed with the intention of expanding functional magnetic resonance imaging (fMRI) capabilities to record brain activity associated with behavioral tasks involving writing and drawing, particularly “paper and pencil” tests used clinically for neuropsychological evaluation of patients. The present work involves initial evaluation of the tablet system applied to transcranial MRgFUS for treatment of ET. The specific goals are: 1) to adapt existing, validated tests for use with the tablet system to assess ET; 2) to establish initial metrics to quantify performance on these tests; 3) to apply the tablet system during MRgFUS treatment of ET; and 4) to demonstrate treatment effects using the tablet system metrics. The quality and ramifications of the resulting data are subsequently discussed, as well as modifications that could be made to the tablet system to enhance its utility for MRgFUS applications in the future.

## Methods

Twelve ET patients (64–85 y.o.; 4 females; 4 left handed) were recruited from the participants of clinical trials of MRgFUS for unilateral thalamotomy (NCT01827904) [5], at the Sunnybrook Health Sciences Centre location. The patients gave separate informed consent to participate in the present pilot study using the tablet, with approval of the local Research Ethics Board. For the pre-operative test, one to two weeks prior to the MRgFUS procedure, participants sat in a chair in front of a computer with the tablet resting in their lap. They first familiarized themselves with the use of the tablet and stylus by writing their name with their unsupported dominant hand (to be treated), before continuing on to spiral and line drawing tasks (described below). They then repeated the same drawing tasks with the non-dominant hand, to serve as a baseline that was not expected to change after the unilateral procedure. As this was a proof-of-concept demonstration, logistical considerations, e.g. length of treatment time and occasional technical issues related to the MRgFUS equipment, dictated that three participants were not tested intra-operatively, but they did complete the pre- and post-operative tests. A further three participants completed only the pre-operative test, but neither intra-operative nor post-operative tests. (Two of these participants ultimately did not meet the inclusion criteria for the MRgFUS trial, and one participant was not tested because of scheduling issues.) Therefore, six of the 12 participants completed the drawing tasks intra-operatively between sonication phases, but only with the dominant hand (contralateral to the ablation). These participants were supine with the tablet supported by cushions on their abdomen, and they wore modified prism glasses (Scan Sound, Inc., Deerfield, FL) to view the computer display on a projection screen located outside the MRI bore. Finally, nine of the 12 participants were tested post-operatively, several minutes after exiting the MRI room and removing the stereotactic frame, following the same procedure as the pre-operative test.

**Fig. 1:**
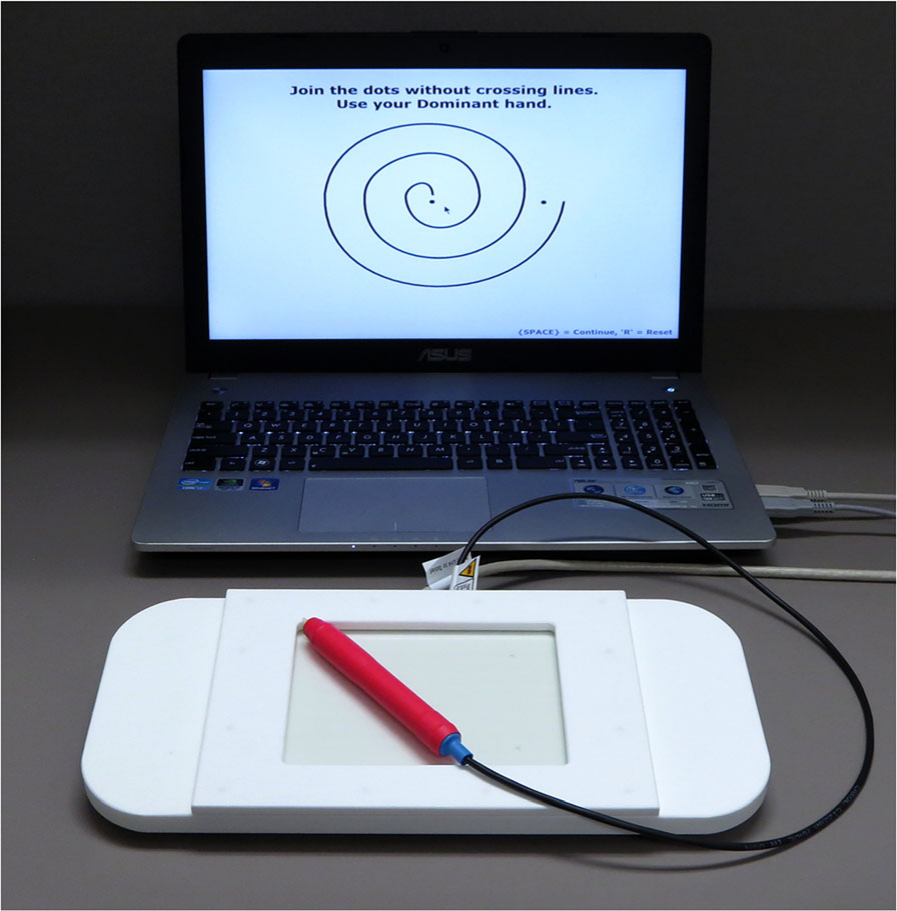
Testing computer and MRI-compatible tablet. The tablet was placed on the participant’s lap when seated for pre- and post-operative tests, and was supported by cushions on the participant’s abdomen for intra-operative testing during MRgFUS

The MRI-compatible touch tablet and stylus (Fig. 1) were connected to a computer running an E-Prime (Psychology Software Tools, Sharpsburg, PA) program that allowed patients to “draw” on screens derived from the drawing section (Part B) of the Fahn-Tolosa-Marin Tremor Rating Scale [6]. This qualitative/semi-quantitative rating scale is widely used in research and clinically, but inter-rater reliability can be poor for the drawing items [9, 10], a disadvantage that may be overcome through use of a tablet [11]. Drawing A (Fig. 3a) was a large spiral with two full turns between guide lines, spaced 10 mm in real terms on the tablet surface. Drawing B (Fig. 3b) was a smaller, tighter spiral with three full turns between guide lines, spaced 5 mm. Drawing C (Fig. 3c) consisted of three separate, straight lines drawn between guide lines spaced 10 mm, 7 mm, and 4 mm. The stylus tip position was logged with 0.1 mm resolution at 600 Hz, and the completed trial screen was also captured for review. Each trial was self-paced, and participants were allowed to repeat trials, for example after clarifying instructions or because they were unable to maintain contact with the tablet during a trial.

**Fig. 2:**
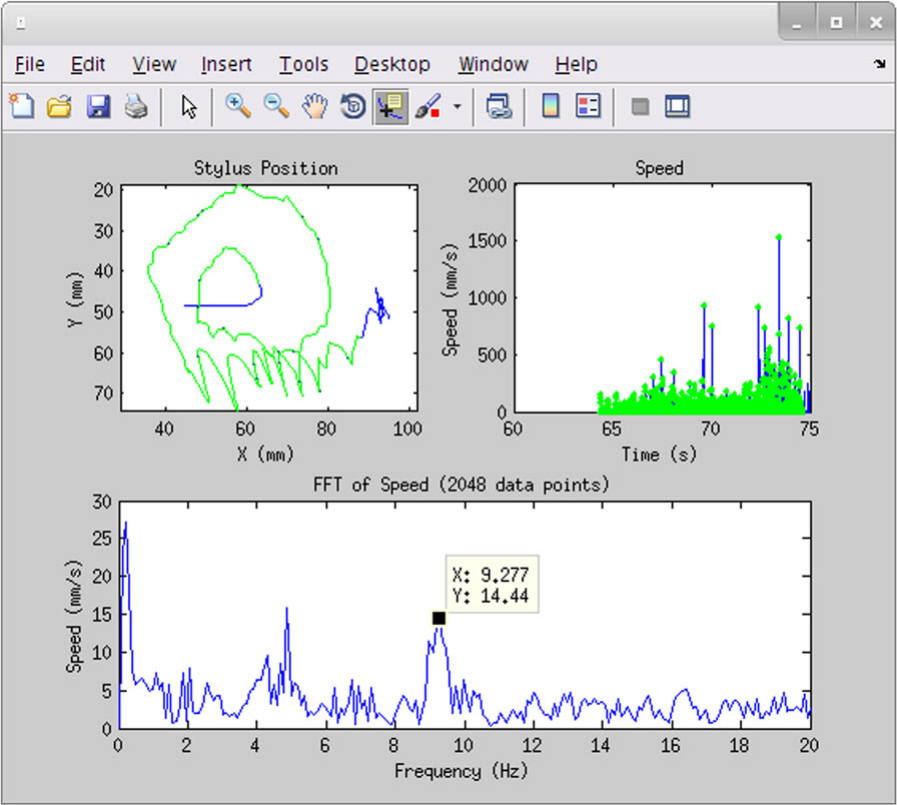
Prototype interface used for data selection and quality inspection. The *upper left plot* shows the raw cursor data (*blue*) and the selected, resampled cursor data (*green overlay*), which can be compared with the drawing screenshot (not shown). The *upper right plot* shows the speed over time (*blue*) and the selected data points (*green dots*). The *lower plot* shows the fast Fourier transform of the selected speed data, from which peaks were chosen for tremor metric calculation

Instead of simply rating the drawings on a semi-quantitative scale as in the original test on paper, the position data logs from the tablet were processed using MATLAB (Mathworks, Natick, MA) to calculate a tremor metric which has been validated in ET patients [12]. The metric’s spectral analysis of stylus speed rather than position makes it relatively easy to calculate and meaningful regardless of the actual stylus trajectory, and it also emphasizes high-frequency tremor relative to low-frequency voluntary movement, in proportion to the frequency [13]. Intuitively, a good performance would have constant or slowly varying speed, while a tremulous performance would have oscillating speed. Position data were trimmed to remove non-response samples; down-sampled to 200 Hz; converted to speed in mm/s; censored to remove long pauses and unusually large values, likely due to lift-and-replace movements or inadvertent tablet contact; and then entered into a 2048-point fast Fourier transform (truncating or zero-padding as needed) to locate spectral power peaks in the 4–16 Hz range. The data were plotted (Fig. 2) at intermediate stages of this process, for inspection of data quality. The area under the curve of the Fourier transform in 2-Hz bands centred on each peak was calculated, and the maximum area under the curve was recorded as the tremor metric for each trial. Finally, Wilcoxon signed rank tests were planned to compare paired pre- vs. post-operative tremor metrics for each drawing type (A, B, C) and hand (Dominant, Non-dominant). Because three drawing types were included to explore their respective utility, α was conservatively defined as 0.05 / 3 = 0.017 for these planned comparisons, and uncorrected *p*-values are presented below.

## Results

**Fig. 3:**
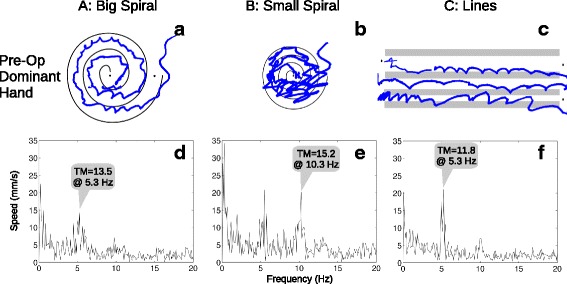
Example pre-operative drawings from a single participant. The three drawings (**a**–**c**) were made using the dominant hand (to be treated). The preprocessed, fast Fourier transformed speed data are plotted (**d**–**f**) below each drawing, with the tremor metric (TM) annotated above the largest peak

**Fig. 4:**
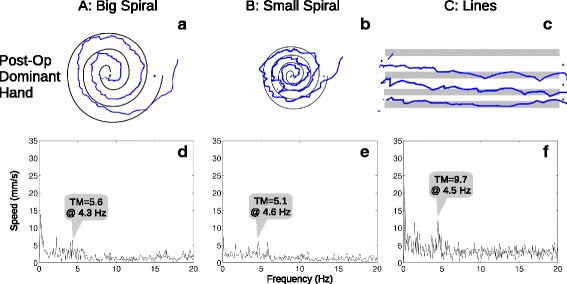
Example post-operative drawings from a single participant. Data are displayed in the same format as in Fig. 3, for the same participant immediately after MRgFUS treatment. In this example, all the post-operative tremor metrics were lower than the pre-operative values

**Fig. 5:**
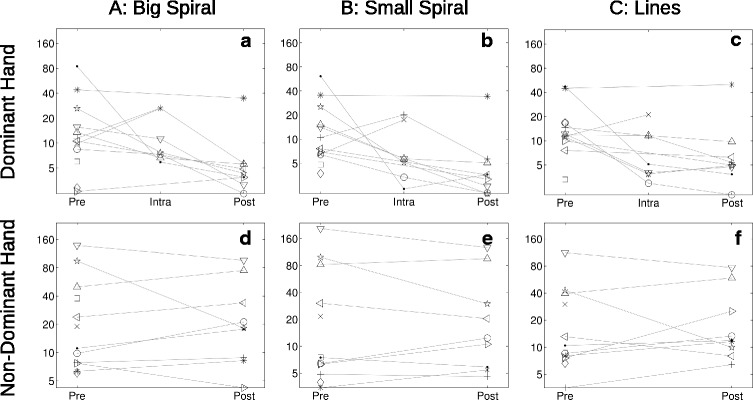
Plots of tremor metrics. Tremor metrics plotted on a logarithmic scale (mm/s^2^), measured at each time point (Pre: pre-operative; Intra: intra-operative; Post: post-operative) with Dominant (**a–c**) and Non-Dominant (**d–f**) hands, for each of the 3 drawing types (*columns*). Each participant’s data points are distinguished by different symbols and joined by lines to help visualize changes. The intra-operative metrics are the mean of one to three separate tests conducted on the MRI scanner table, generally between ablative sonications later in the procedure

Examples of the three drawing types and plots of the associated Fourier transforms are shown in Figs. 3 and 4 for one participant (pre- and post-operative, respectively, dominant hand only). All participants’ tremor metrics for each drawing type and hand are plotted over time in Fig. 5. For the planned comparisons, eight participants’ data were used, after dropping one participant because the MRgFUS procedure had terminated without successful ablation due to a technical issue. Significant (*p* < 0.016, uncorrected) pre- vs. post-operative decreases were seen in the tremor metric calculated for all three drawing types in the dominant (treated) hand, but the differences were not significant in the non-dominant (untreated) hand, as summarized in Table 1 along with descriptive statistics. Additionally, intra-operative metrics tended to be intermediate between the pre- and post-operative metrics, with median values of 7.5, 5.4, and 4.6 mm/s^2^ for Drawings A, B, and C, respectively (dominant hand only).

**Table 1:** Statistics of tremor metrics

	Drawing A: Big Spiral		Drawing B: Small Spiral		Drawing C: Lines	
	Pre	Post	Pre	Post	Pre	Post
Dominant Hand	12.5	4.1	12.3	3.4	12.0	4.9
	10.0–18.3	3.7–5.1	7.4–17.7	2.5–4.0	10.9–15.2	4.5–5.6
	***p*** **= 0.016** ^*^		***p*** **= 0.008** ^*^		***p*** **= 0.008** ^*^	
Non-Dominant Hand	17.5	19.7	18.9	16.4	11.8	12.6
	9.3–61.1	15.6–44.1	6.4–86.5	9.4–46.3	8.3–40.4	9.4–33.5
	*p* = 0.945		*p* = 0.547		*p* = 0.945	

Descriptive statistics and test results on tremor metrics (mm/s^2^) for each drawing type and hand, at pre- and post-operative time points. The first row in each Drawing x Hand cell contains the median tremor metrics; the second row contains the first–third quartile range; and the third row is the result of the pre- vs. post-operation tremor metric comparison, which is shown in bold numbers and marked with an asterisk if significant (α = 0.017, *p*-values uncorrected)

## Discussion

This study demonstrates an MRI-compatible setup for quantitative tremor measurement that can be used pre-, post-, and intra-operatively during MRgFUS thalamotomy procedures, for treatment monitoring and to inform neurosurgical decision-making. Drawing tasks from the widely-used Fahn-Tolosa-Marin Tremor Rating Scale were adapted as faithfully as possible, and a validated quantitative tremor metric [12] was calculated. The system performed as expected, with the results reflecting a significant decrease in tremor of the dominant (treated) hand, but not the non-dominant (untreated) hand. The results are highly suggestive of the MRgFUS treatment’s short-term efficacy. However, to be clear, the present pilot study was not designed to examine the treatment’s value—there was no control group, for example. Instead, we showed the utility of the MRI-compatible tablet system for tremor quantification during various stages of MRgFUS treatment.

A major feature of the tablet system is that it enables quantitative tremor measurement during the procedure in the MRI suite. Currently, the MRgFUS procedure can be quite lengthy, depending largely on the difficulty of localization and occasionally due to technical issues, so our proof-of-concept study was assigned a low priority, and for a few patients the decision was made to omit the tablet experiment. No attempt at hypothesis testing was planned or attempted with the six participants who had varying numbers of intra-operative tests with the tablet. Nevertheless, intra-operative metrics tended to be intermediate between pre- and post-operative metrics in the dominant (treated) hand. The intra-operative tremor values were closer to the post-operative values, perhaps because the intra-operative tests occurred later in the procedure, during the longer cool-down periods between high-energy ablative sonications. On the other hand, the intra-operative tests were performed while lying down, and this difference in posture means these tests may not be directly comparable to the pre- and post-operative tests. Additional testing would be required to explore how the metrics differ when lying down versus sitting. However, such a difference would not preclude use of this test on the scanner table, because in this context the test’s purpose is to quantify change from a baseline acquired early in the procedure. Further testing with more deliberate intra-operative testing is suggested to examine how test performance changes during the MRgFUS procedure, and how such changes relate to clinical outcomes. This knowledge could potentially aid treatment decision-making by establishing treatment optimization thresholds based on quantitative tremor measurements (e.g. terminating treatment after reaching X% tremor reduction, or after reaching some plateau). However, such a scheme should ideally be a joint optimization with yet-to-be determined measurements of adverse effects, which still occur at substantial levels in early ET MRgFUS trials [5].

After testing the three different drawing types, no strong conclusions can be made about their relative usefulness. The tremor metrics for all three drawings were fairly consistent: 7 of 8 participants showed a pre-post decrease in Drawing A, whereas 8 of 8 participants showed a decrease in Drawings B and C. However, some minor practical issues were observed when participants were tested with Drawing C. Some participants drew the straight lines with quick, “ballistic” movements, rather than deliberate and well-controlled strokes. They were asked to redo the test in extreme cases, but this issue may still be true to some degree. This type of motion is not the sort expected to generate the most tremor, and it shortens the sampling time, thus reducing the accuracy of the measurement. This may have contributed to the lower median tremor metrics for Drawing C in the non-dominant hand. It was also not obvious to users, without clear instructions, whether the lines should be drawn separately and in which direction. These issues are not evident with the spirals, and furthermore spirals have more literature validating their use with quantification [11–14]. The added time required to complete all three drawings rather than one or two is minimal, however. All three drawings should be performed for completeness, with additional direction given to ensure the consistency of the drawing technique in Drawing C.

Future practical enhancements of the testing setup include improved automation of stylus data censoring and selection, to enable quicker, interactive processing. Flexibility is important because, across participants, there was quite a lot of variability in start/end times and locations, speed, and severity of tremor, which made it challenging to select portions of drawings automatically where the participants were clearly on-task. Our MATLAB-based prototype interface (Fig. 2) was a step towards ensuring data quality, but it could be augmented to allow interactive selection of the various censorship options, the data for inclusion, and peak frequencies for tremor metric calculation. Another potentially useful enhancement is the inclusion of stylus pressure in the data analysis, which increases complexity but has been shown to reflect tremor severity [14]. In the present work, we recorded pressure but did not use it in the absence of clear instructions or meaningful feedback to the participants. The drawing tasks could be augmented to change the size and/or color of the line or cursor when the participant moves out of a target range, analogous to the spiral guide lines in the X-Y plane. The subsequent data analysis could then act on the pressure data separately or in combination with the X-Y coordinates. Additionally, accelerometers and electromyography have been used to quantify tremor in research with drawing tasks as well as other action tasks and rest [15]. With the commercial availability of MRI-compatible accelerometers and electromyographs, quantification of many of the other items of the Fahn-Tolosa-Marin Tremor Rating Scale is now possible. Finally, the patient’s sight lines in the current MRgFUS setup are highly constrained by the transducer array “helmet”, the attached membrane that bulges out over the eyes, and anterior elements of the stereotactic frame. A modified pair of prism glasses were used in the present work, mounted above the eyes with tape, to allow participants to see around these obstructions. For some participants the glasses were positioned only temporarily for the duration of each intra-operative test, to prevent any influence of the glasses on the ultrasound transmission due to contact with the membrane. An articulated mount will be constructed to make it easier and quicker to position the glasses.

With these refinements, MRI-compatible tablet-based tremor quantification during MRgFUS thalamotomy has potential to improve clinical trials by increasing their precision and objectivity. With additional work, this may eventually lead to improved patient outcomes both indirectly, through improved research quality, and directly, by providing a metric for optimization during the operation to maximize treatment effect, in concert with other tests aimed at minimizing adverse effects. Reduced costs may result both indirectly, through improved patient outcomes and reduced followup treatment, and directly, by saving time with automated testing that can be initiated without entering the MRI suite and repositioning the patient table, possibly even occurring during sonication. Alternatively, the time saved could be used to measure tremor more frequently for a finer grained appreciation of the change during the procedure, and it could be used to do more thorough testing for adverse effects. The apparatus may also be of use during fMRI of ET and other diseases with tremor such as Parkinson’s disease (PD), although this may require particularly effective head motion correction strategies when not using a stereotactic frame. It remains to be seen whether fMRI using the present drawing task would be of value in localization of the MRgFUS treatment volume. There is also increasing evidence that, like PD, ET is associated with a range of neurocognitive deficits [16], and the tablet can enable study of the neurological bases of these deficits, using standard paper-and-pencil neuropsychological tests during fMRI.

## Conclusions

An MRI-compatible setup for quantitative tremor measurement was demonstrated that can be used pre-, post-, and intra-operatively during MRgFUS thalamotomy procedures, complementing standard qualitative assessment. After suggested enhancements and further testing, the system has potential to add objectivity to clinical trials and may lead to more optimal treatment. Extensions to fMRI studies of ET and PD are also likely.

## Abbreviations

ET: Essential tremor
fMRI: Functional magnetic resonance imaging
MRgFUS: Magnetic resonance-guided focused ultrasound
MRI: Magnetic resonance imaging
PD: Parkinson’s disease
